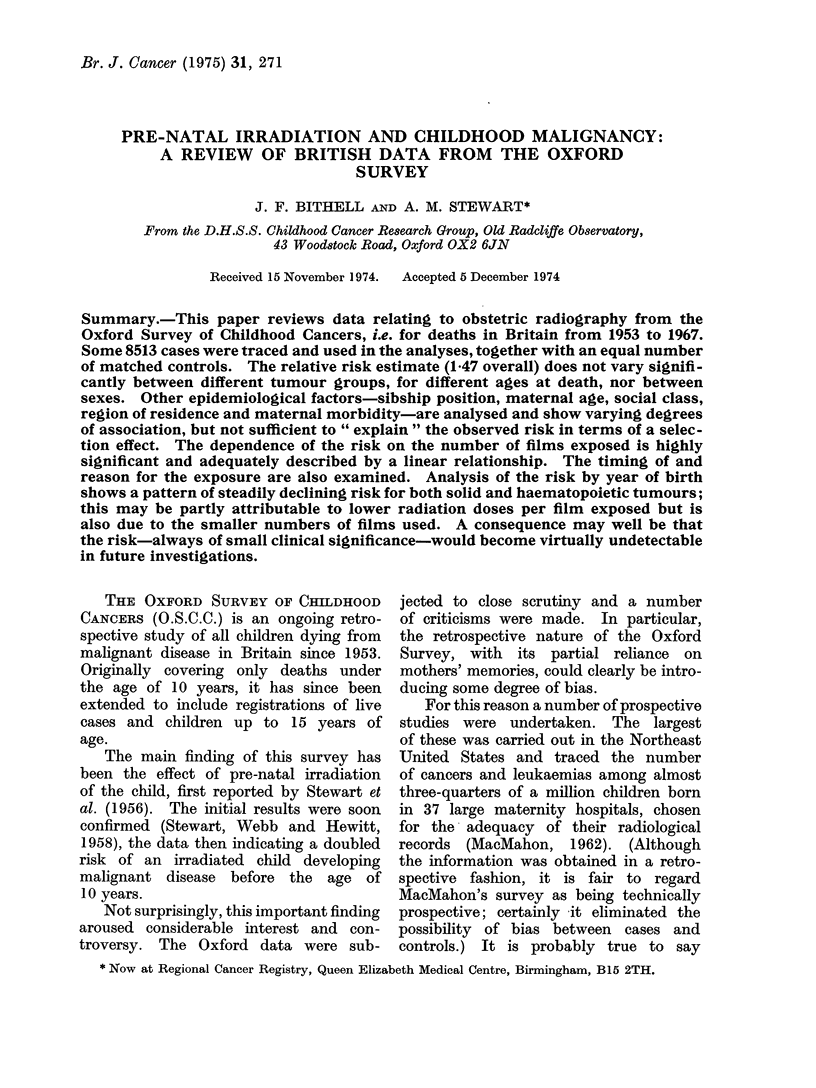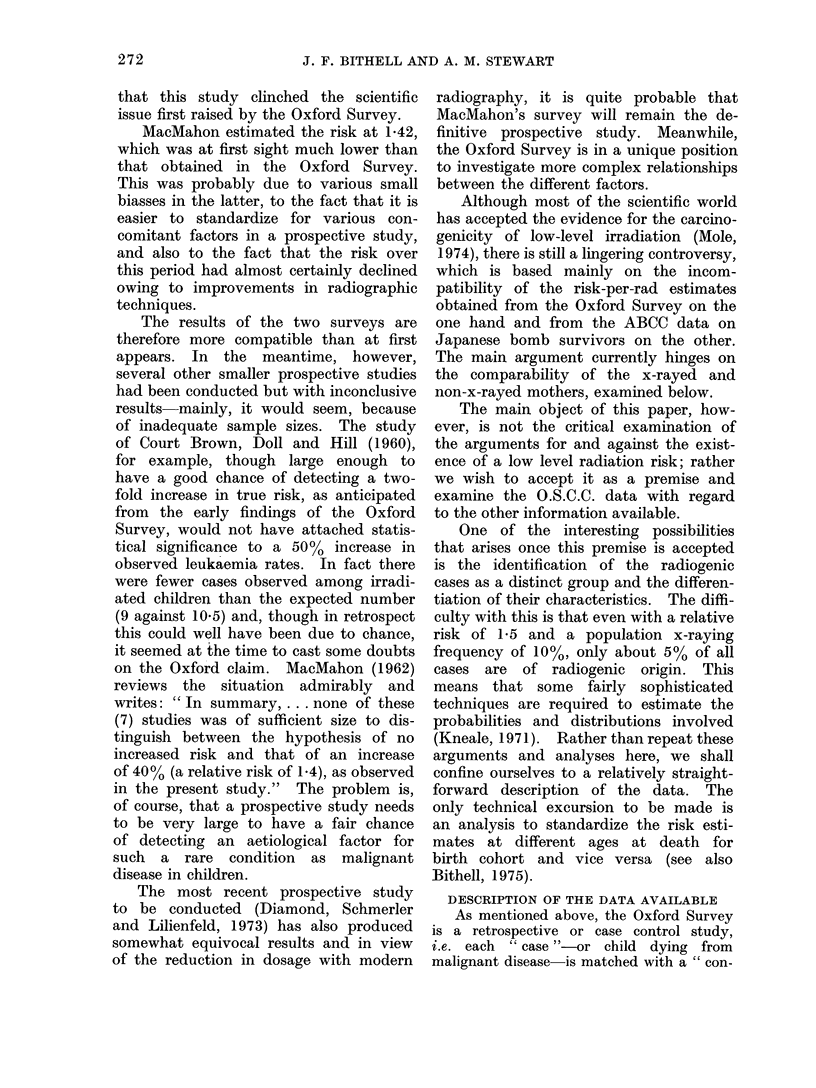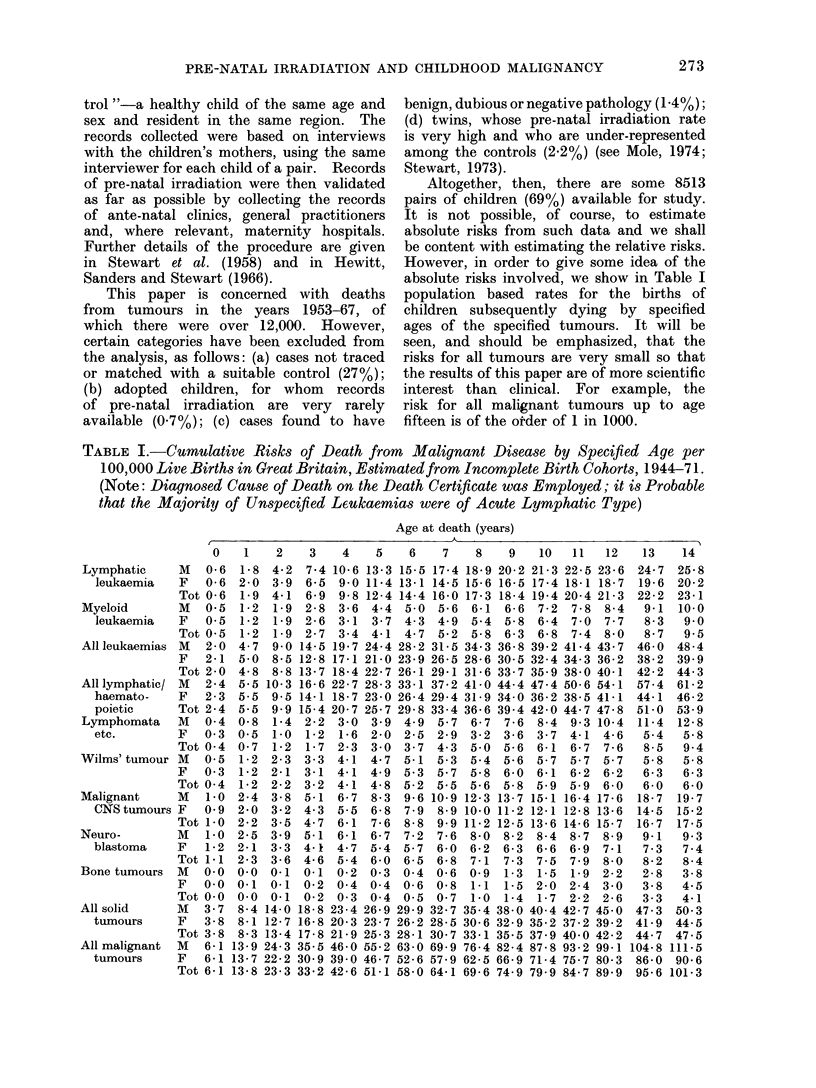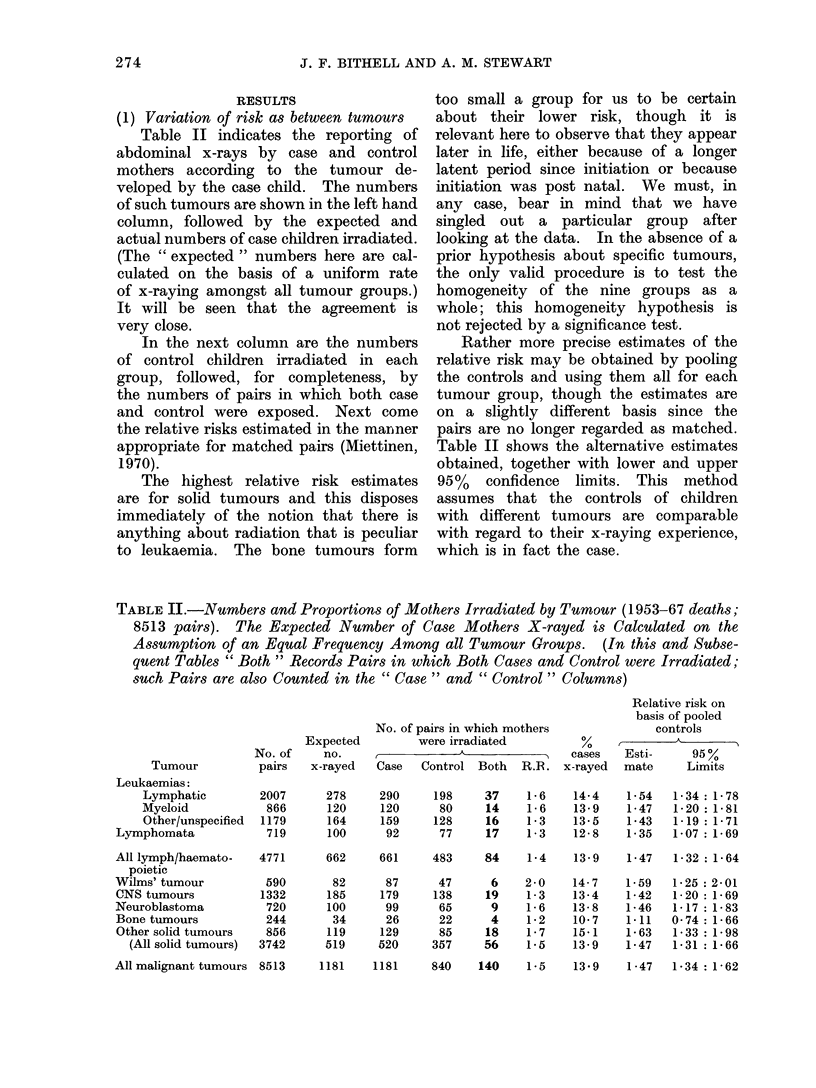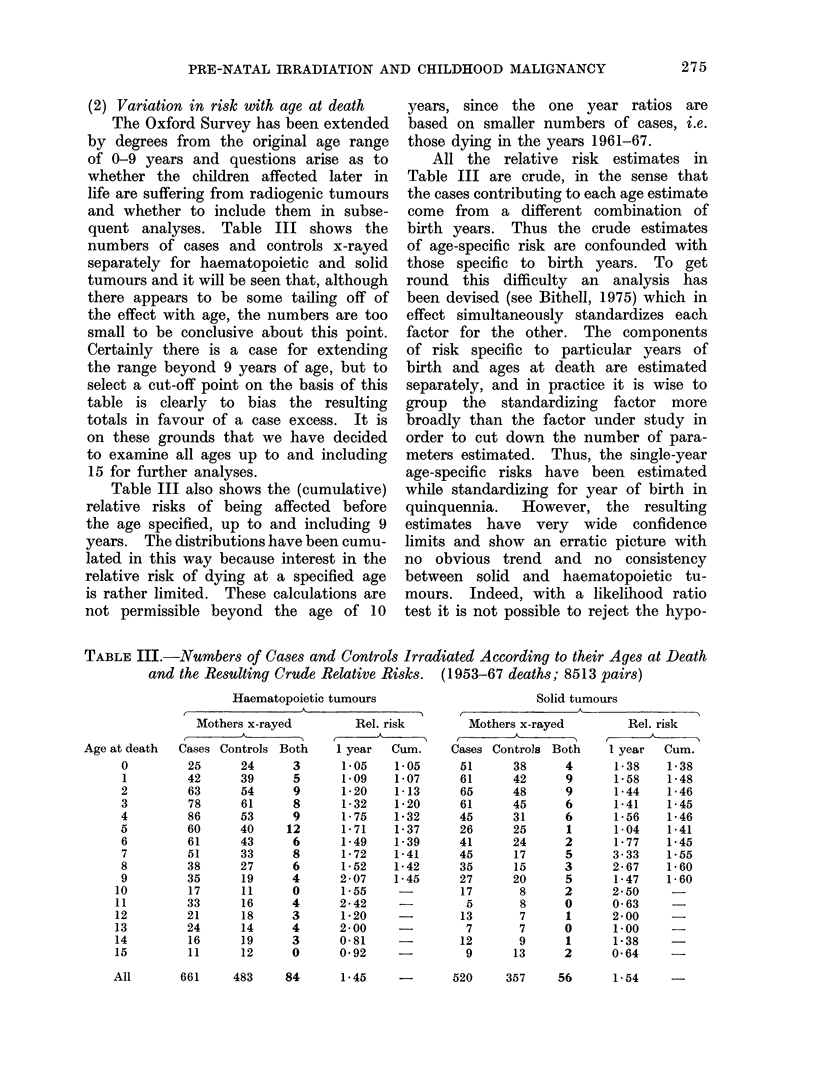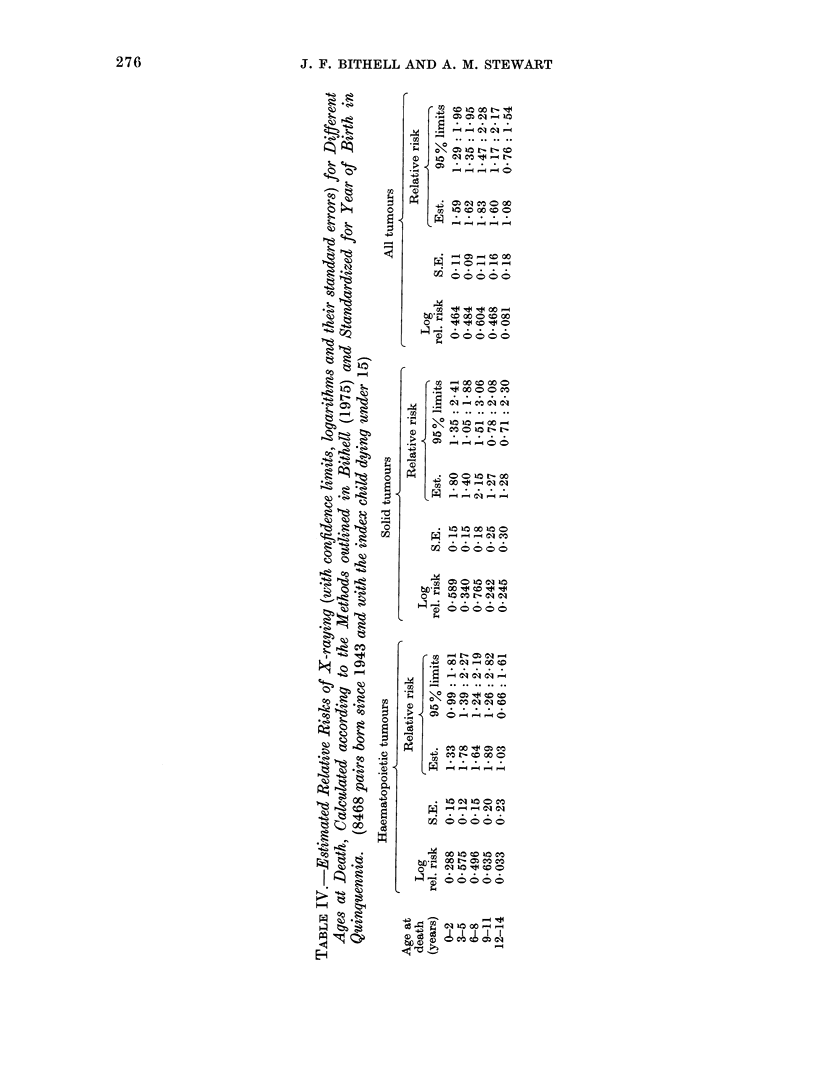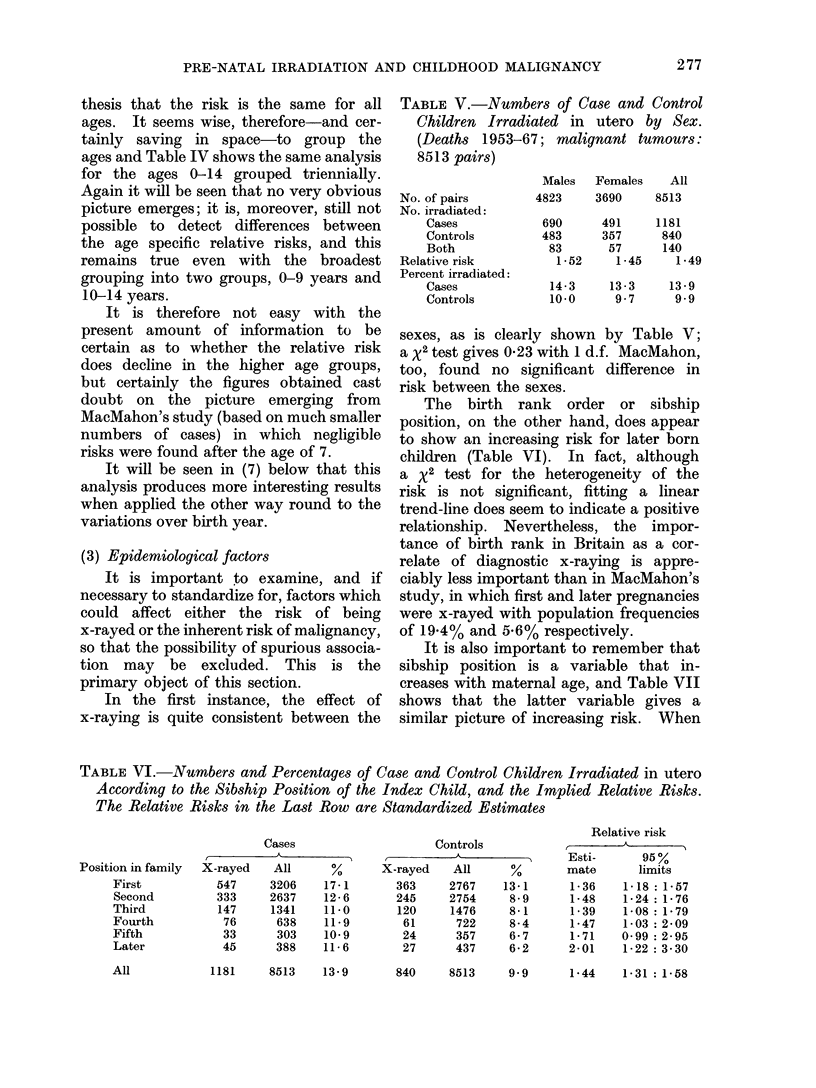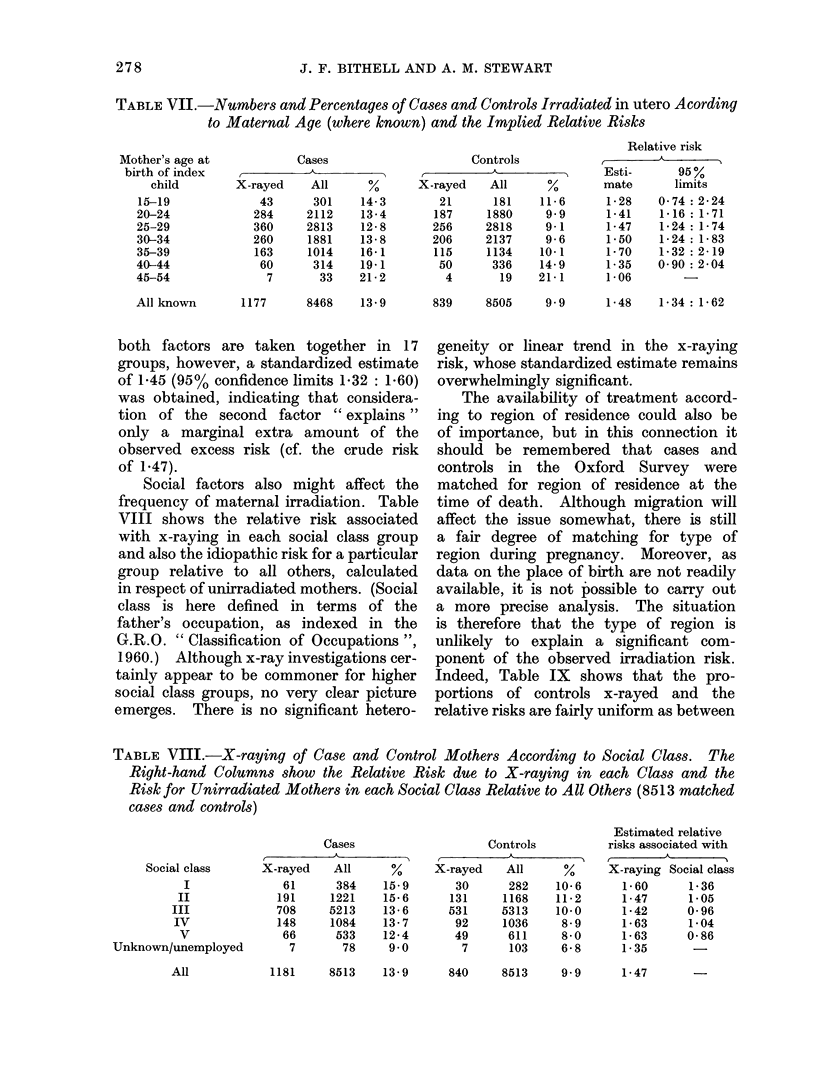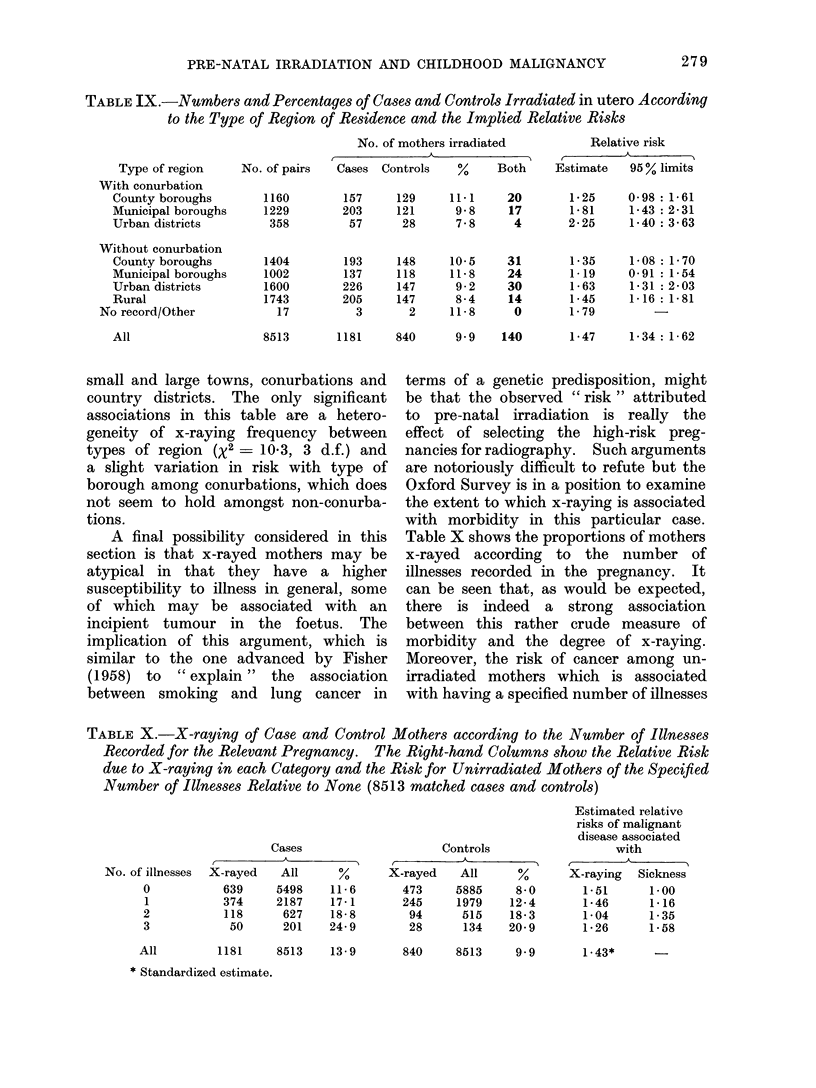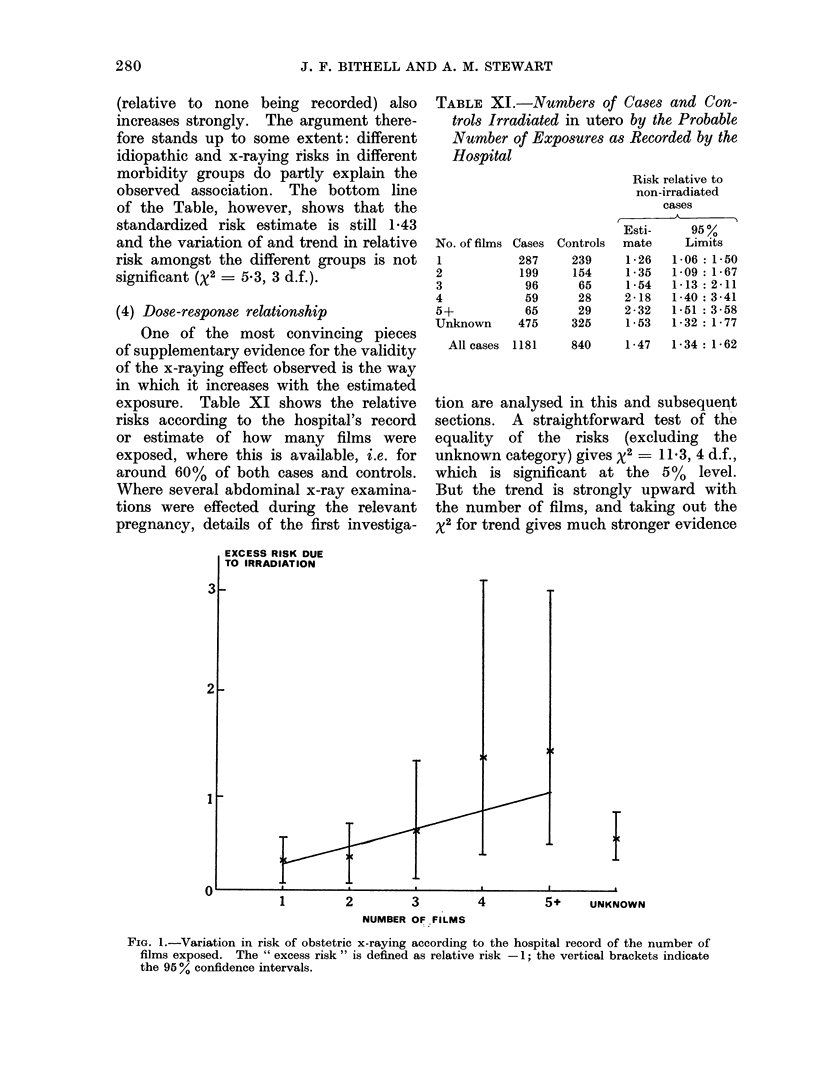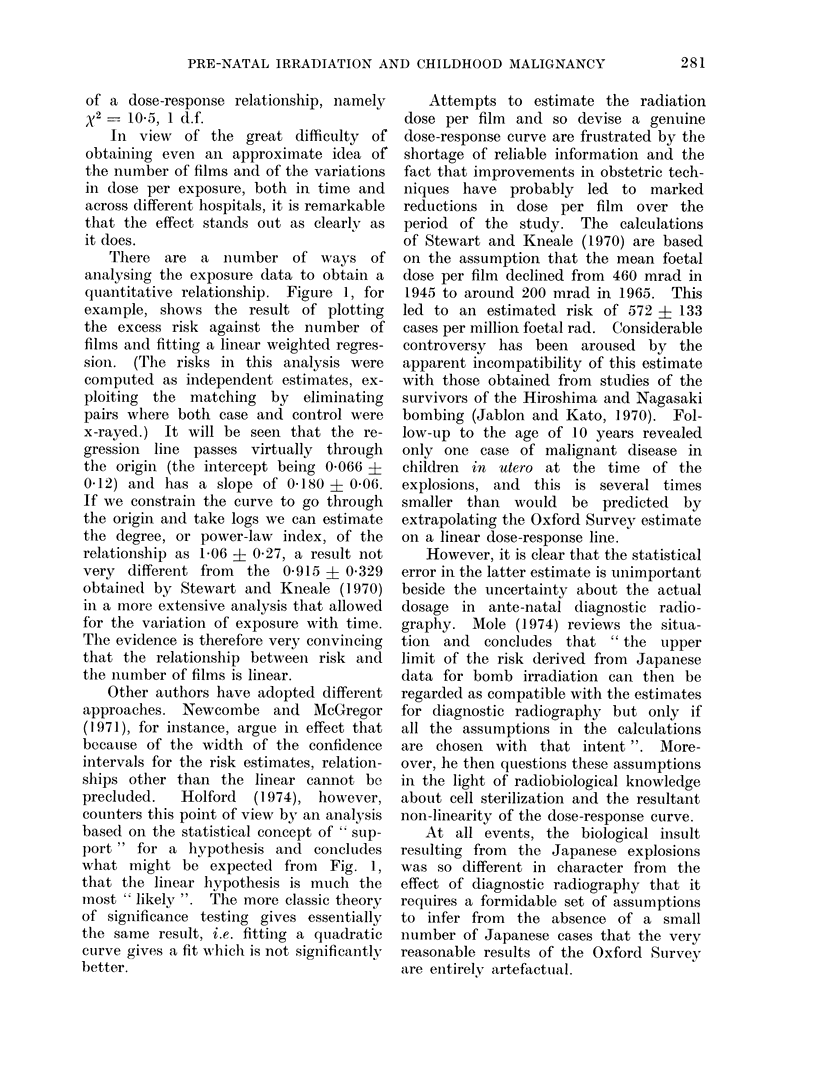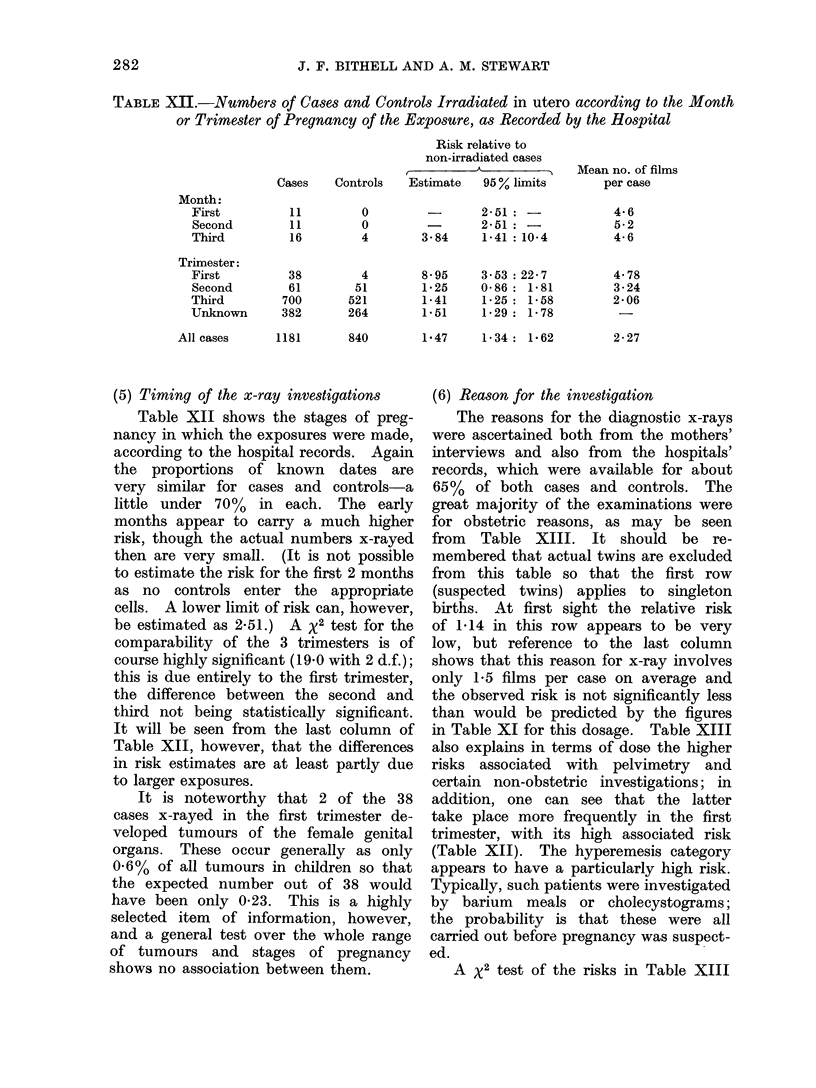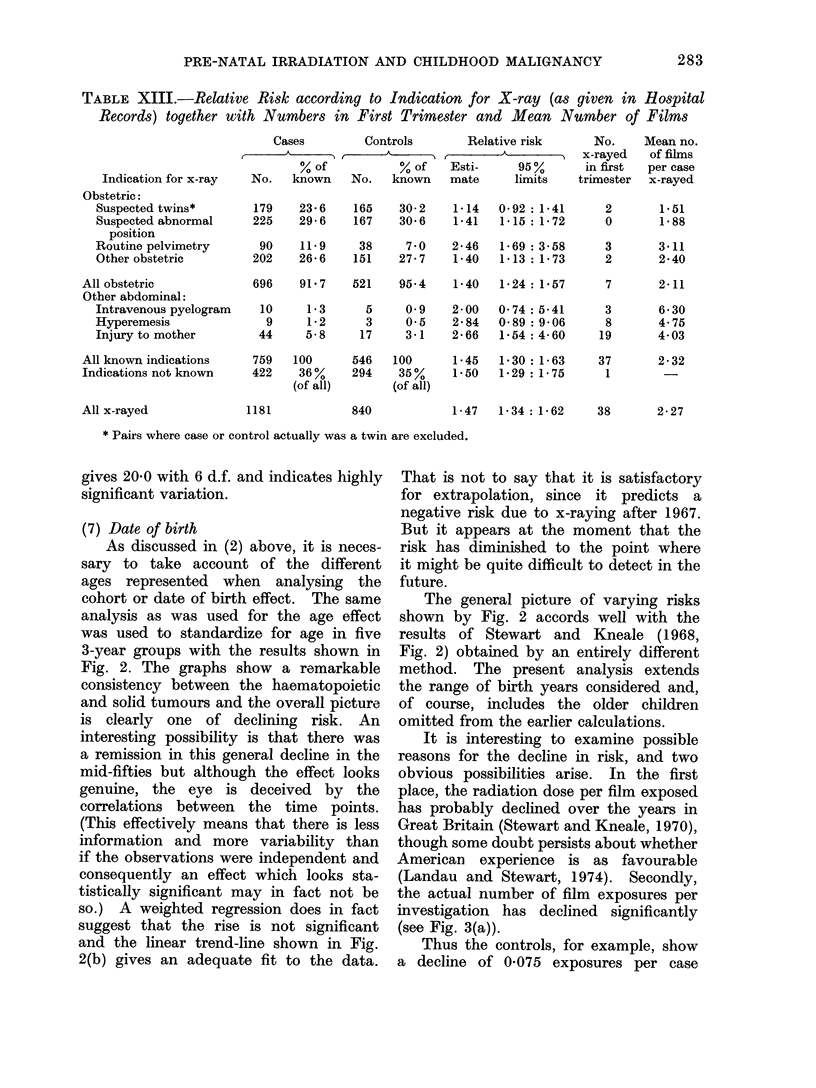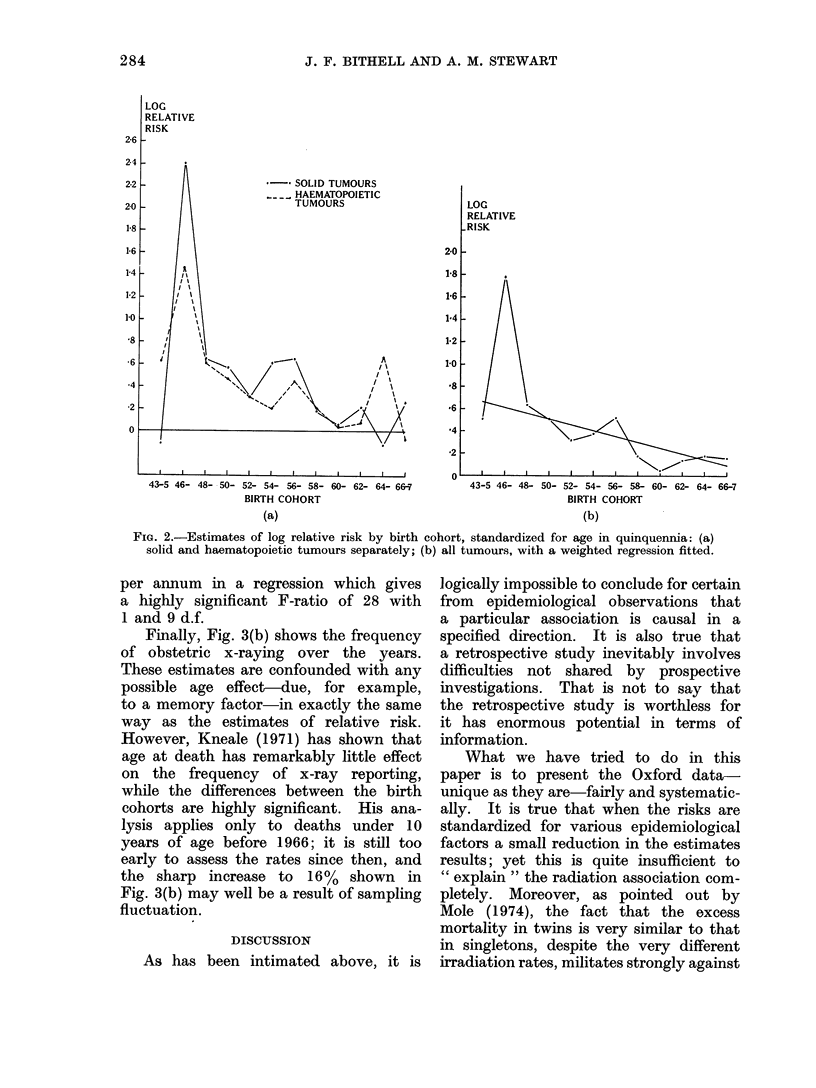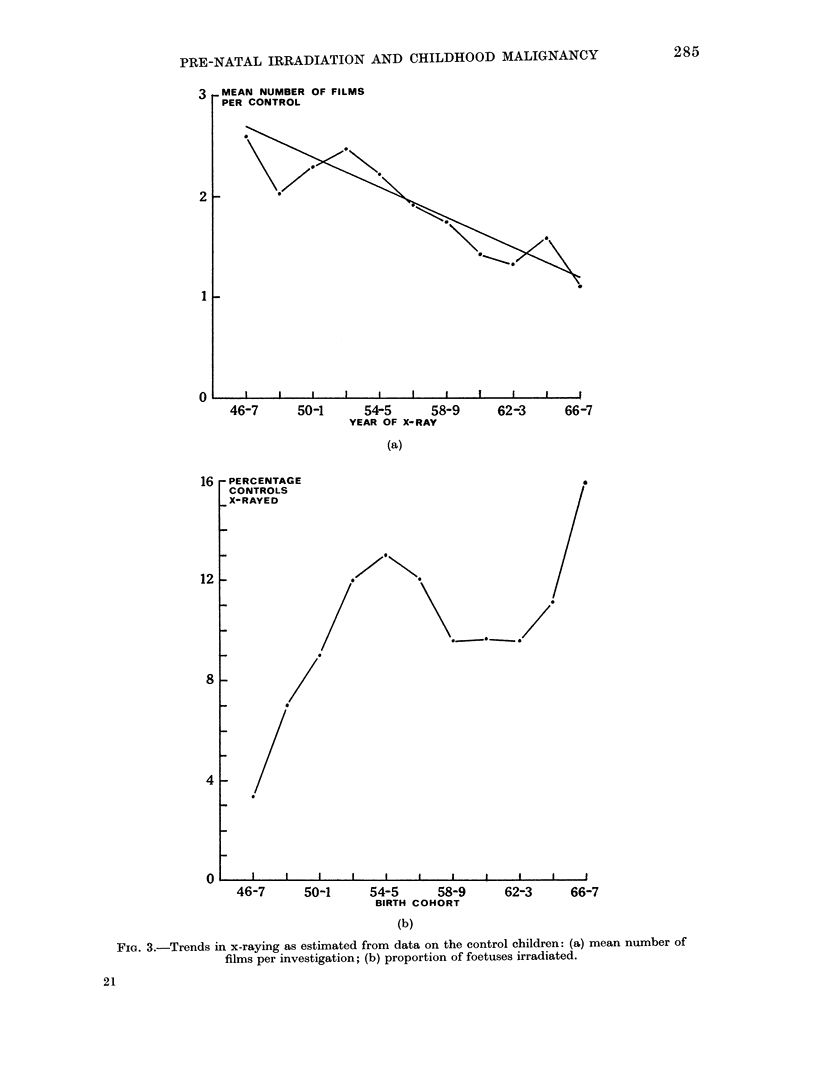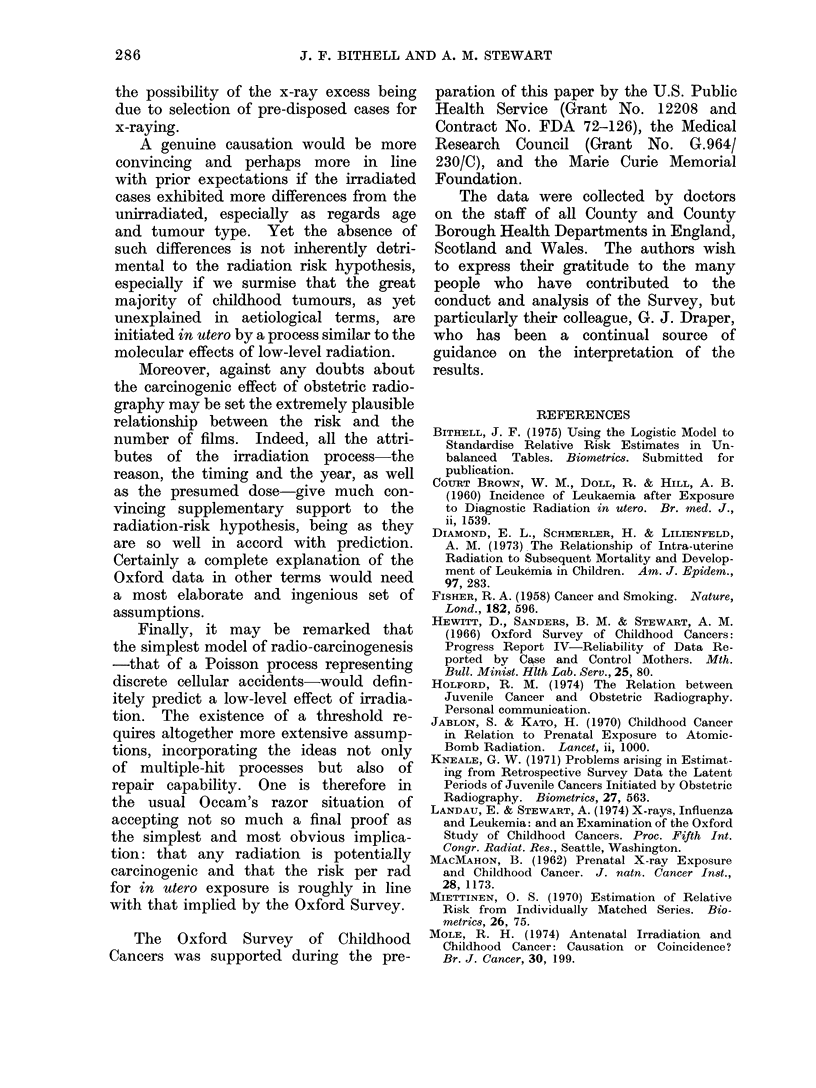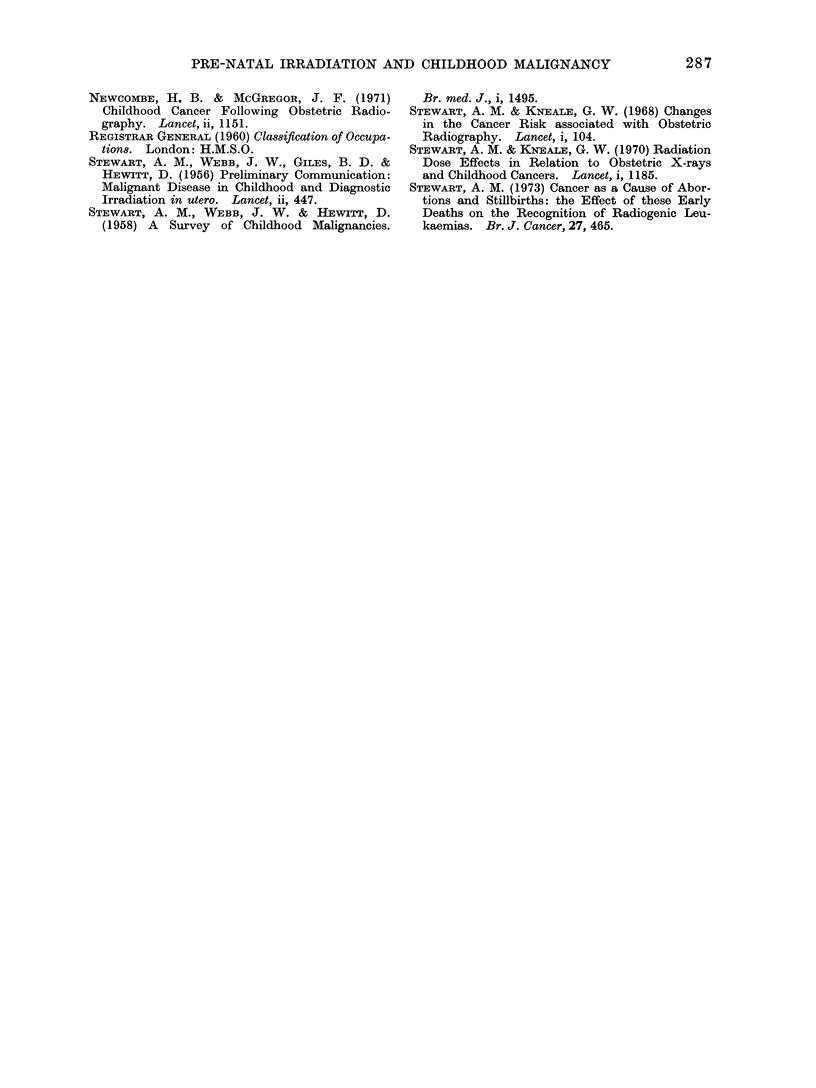# Pre-natal irradiation and childhood malignancy: a review of British data from the Oxford Survey.

**DOI:** 10.1038/bjc.1975.62

**Published:** 1975-03

**Authors:** J. F. Bithell, A. M. Stewart

## Abstract

This paper reviews data relating to obstetric radiography from the Oxford Survey of Childhood Cancers, i.e. for deaths in Britain from 1953 to 1967. Some 8513 cases were traced and used in the analyses, together with an equal number of matched controls. The relative risk estimate (1-47 overall) does not vary significantly between different tumour groups, for different ages at death, nor between sexes. Other epidemiological factors-sibship position, maternal age, social class, region of residence and maternal morbidity-are analysed and show varying degrees of association, but not sufficient to "explain" the observed risk in terms of a selection effect. The dependence of the risk on the number of films exposed is highly significant and adequately described by a linear relationship. The timing of and reason for the exposure are also examined. Analysis of the risk by year of birth shows a pattern of steadily declining risk for both solid and haematopoietic tumours; this may be partly attributable to lower radiation doses per film exposed but is also due to the smaller numbers of films used. A consequence may well be that the risk-always of small clinical significance-would become virtually undetectable in future investigations.


*Br. J. Cancer* (1975) **31**, 271

### PRE-NATAL IRRADIATION AND CHILDHOOD MALIGNANCY: A REVIEW OF BRITISH DATA FROM THE OXFORD SURVEY

J. F. BITHELL AND A. M. STEWART*

*From the D.H.S.S. Childhood Cancer Research Group, Old Radcliffe Observatory, 43 Woodstock Road, Oxford OX2 6JN*

Received 15 November 1974. Accepted 5 December 1974

**Summary.—This paper reviews data relating to obstetric radiography from the Oxford Survey of Childhood Cancers, *i.e.* for deaths in Britain from 1953 to 1967. Some 8513 cases were traced and used in the analyses, together with an equal number of matched controls. The relative risk estimate (1·47 overall) does not vary significantly between different tumour groups, for different ages at death, nor between sexes. Other epidemiological factors—sibship position, maternal age, social class, region of residence and maternal morbidity—are analysed and show varying degrees of association, but not sufficient to " explain " the observed risk in terms of a selection effect. The dependence of the risk on the number of films exposed is highly significant and adequately described by a linear relationship. The timing of and reason for the exposure are also examined. Analysis of the risk by year of birth shows a pattern of steadily declining risk for both solid and haematopoietic tumours; this may be partly attributable to lower radiation doses per film exposed but is also due to the smaller numbers of films used. A consequence may well be that the risk—always of small clinical significance—would become virtually undetectable in future investigations.**

THE OXFORD SURVEY OF CHILDHOOD CANCERS (O.S.C.C.) is an ongoing retrospective study of all children dying from malignant disease in Britain since 1953. Originally covering only deaths under the age of 10 years, it has since been extended to include registrations of live cases and children up to 15 years of age.

The main finding of this survey has been the effect of pre-natal irradiation of the child, first reported by Stewart *et al.* (1956). The initial results were soon confirmed (Stewart, Webb and Hewitt, 1958), the data then indicating a doubled risk of an irradiated child developing malignant disease before the age of 10 years.

Not surprisingly, this important finding aroused considerable interest and controversy. The Oxford data were subjected to close scrutiny and a number of criticisms were made. In particular, the retrospective nature of the Oxford Survey, with its partial reliance on mothers' memories, could clearly be introducing some degree of bias.

For this reason a number of prospective studies were undertaken. The largest of these was carried out in the Northeast United States and traced the number of cancers and leukaemias among almost three-quarters of a million children born in 37 large maternity hospitals, chosen for the adequacy of their radiological records (MacMahon, 1962). (Although the information was obtained in a retrospective fashion, it is fair to regard MacMahon's survey as being technically prospective; certainly it eliminated the possibility of bias between cases and controls.) It is probably true to say

* Now at Regional Cancer Registry, Queen Elizabeth Medical Centre, Birmingham, B15 2TH.

that this study clinched the scientific issue first raised by the Oxford Survey.

MacMahon estimated the risk at 1·42, which was at first sight much lower than that obtained in the Oxford Survey. This was probably due to various small biasses in the latter, to the fact that it is easier to standardize for various concomitant factors in a prospective study, and also to the fact that the risk over this period had almost certainly declined owing to improvements in radiographic techniques.

The results of the two surveys are therefore more compatible than at first appears. In the meantime, however, several other smaller prospective studies had been conducted but with inconclusive results—mainly, it would seem, because of inadequate sample sizes. The study of Court Brown, Doll and Hill (1960), for example, though large enough to have a good chance of detecting a twofold increase in true risk, as anticipated from the early findings of the Oxford Survey, would not have attached statistical significance to a 50% increase in observed leukaemia rates. In fact there were fewer cases observed among irradiated children than the expected number (9 against 10·5) and, though in retrospect this could well have been due to chance, it seemed at the time to cast some doubts on the Oxford claim. MacMahon (1962) reviews the situation admirably and writes: "In summary, . . . none of these (7) studies was of sufficient size to distinguish between the hypothesis of no increased risk and that of an increase of 40% (a relative risk of 1·4), as observed in the present study." The problem is, of course, that a prospective study needs to be very large to have a fair chance of detecting an aetiological factor for such a rare condition as malignant disease in children.

The most recent prospective study to be conducted (Diamond, Schmerler and Lilienfeld, 1973) has also produced somewhat equivocal results and in view of the reduction in dosage with modern radiography, it is quite probable that MacMahon's survey will remain the definitive prospective study. Meanwhile, the Oxford Survey is in a unique position to investigate more complex relationships between the different factors.

Although most of the scientific world has accepted the evidence for the carcinogenicity of low-level irradiation (Mole, 1974), there is still a lingering controversy, which is based mainly on the incompatibility of the risk-per-rad estimates obtained from the Oxford Survey on the one hand and from the ABCC data on Japanese bomb survivors on the other. The main argument currently hinges on the comparability of the x-rayed and non-x-rayed mothers, examined below.

The main object of this paper, however, is not the critical examination of the arguments for and against the existence of a low level radiation risk; rather we wish to accept it as a premise and examine the O.S.C.C. data with regard to the other information available.

One of the interesting possibilities that arises once this premise is accepted is the identification of the radiogenic cases as a distinct group and the differentiation of their characteristics. The difficulty with this is that even with a relative risk of 1·5 and a population x-raying frequency of 10%, only about 5% of all cases are of radiogenic origin. This means that some fairly sophisticated techniques are required to estimate the probabilities and distributions involved (Kneale, 1971). Rather than repeat these arguments and analyses here, we shall confine ourselves to a relatively straightforward description of the data. The only technical excursion to be made is an analysis to standardize the risk estimates at different ages at death for birth cohort and vice versa (see also Bithell, 1975).

### DESCRIPTION OF THE DATA AVAILABLE

As mentioned above, the Oxford Survey is a retrospective or case control study, *i.e.* each "case"—or child dying from malignant disease—is matched with a "control "—a healthy child of the same age and sex and resident in the same region. The records collected were based on interviews with the children's mothers, using the same interviewer for each child of a pair. Records of pre-natal irradiation were then validated as far as possible by collecting the records of ante-natal clinics, general practitioners and, where relevant, maternity hospitals. Further details of the procedure are given in Stewart *et al.* (1958) and in Hewitt, Sanders and Stewart (1966).

This paper is concerned with deaths from tumours in the years 1953–67, of which there were over 12,000. However, certain categories have been excluded from the analysis, as follows: (a) cases not traced or matched with a suitable control (27%); (b) adopted children, for whom records of pre-natal irradiation are very rarely available (0·7%); (c) cases found to have benign, dubious or negative pathology (1·4%); (d) twins, whose pre-natal irradiation rate is very high and who are under-represented among the controls (2·2%) (see Mole, 1974; Stewart, 1973).

Altogether, then, there are some 8513 pairs of children (69%) available for study. It is not possible, of course, to estimate absolute risks from such data and we shall be content with estimating the relative risks. However, in order to give some idea of the absolute risks involved, we show in Table I population based rates for the births of children subsequently dying by specified ages of the specified tumours. It will be seen, and should be emphasized, that the risks for all tumours are very small so that the results of this paper are of more scientific interest than clinical. For example, the risk for all malignant tumours up to age fifteen is of the order of 1 in 1000.

TABLE I.—*Cumulative Risks of Death from Malignant Disease by Specified Age per* 100,000 *Live Births in Great Britain, Estimated from Incomplete Birth Cohorts,* 1944–71. (Note: *Diagnosed Cause of Death on the Death Certificate was Employed; it is Probable that the Majority of Unspecified Leukaemias were of Acute Lymphatic Type*)

| | | Age at death (years) | | | | | | | | | | | | | | |
|---|---|---|---|---|---|---|---|---|---|---|---|---|---|---|---|---|
| | | 0 | 1 | 2 | 3 | 4 | 5 | 6 | 7 | 8 | 9 | 10 | 11 | 12 | 13 | 14 |
| Lymphatic leukaemia | M | 0·6 | 1·8 | 4·2 | 7·4 | 10·6 | 13·3 | 15·5 | 17·4 | 18·9 | 20·2 | 21·3 | 22·5 | 23·6 | 24·7 | 25·8 |
| | F | 0·6 | 2·0 | 3·9 | 6·5 | 9·0 | 11·4 | 13·1 | 14·5 | 15·6 | 16·5 | 17·4 | 18·1 | 18·7 | 19·6 | 20·2 |
| | Tot | 0·6 | 1·9 | 4·1 | 6·9 | 9·8 | 12·4 | 14·4 | 16·0 | 17·3 | 18·4 | 19·4 | 20·4 | 21·3 | 22·2 | 23·1 |
| Myeloid leukaemia | M | 0·5 | 1·2 | 1·9 | 2·8 | 3·6 | 4·4 | 5·0 | 5·6 | 6·1 | 6·6 | 7·2 | 7·8 | 8·4 | 9·1 | 10·0 |
| | F | 0·5 | 1·2 | 1·9 | 2·6 | 3·1 | 3·7 | 4·3 | 4·9 | 5·4 | 5·8 | 6·4 | 7·0 | 7·7 | 8·3 | 9·0 |
| | Tot | 0·5 | 1·2 | 1·9 | 2·7 | 3·4 | 4·1 | 4·7 | 5·2 | 5·8 | 6·3 | 6·8 | 7·4 | 8·0 | 8·7 | 9·5 |
| All leukaemias | M | 2·0 | 4·7 | 9·0 | 14·5 | 19·7 | 24·4 | 28·2 | 31·5 | 34·3 | 36·8 | 39·2 | 41·4 | 43·7 | 46·0 | 48·4 |
| | F | 2·1 | 5·0 | 8·5 | 12·8 | 17·1 | 21·0 | 23·9 | 26·5 | 28·6 | 30·5 | 32·4 | 34·3 | 36·2 | 38·2 | 39·9 |
| | Tot | 2·0 | 4·8 | 8·8 | 13·7 | 18·4 | 22·7 | 26·1 | 29·1 | 31·6 | 33·7 | 35·9 | 38·0 | 40·1 | 42·2 | 44·3 |
| All lymphatic/ haemato- poietic | M | 2·4 | 5·5 | 10·3 | 16·6 | 22·7 | 28·3 | 33·1 | 37·2 | 41·0 | 44·4 | 47·4 | 50·6 | 54·1 | 57·4 | 61·2 |
| | F | 2·3 | 5·5 | 9·5 | 14·1 | 18·7 | 23·0 | 26·4 | 29·4 | 31·9 | 34·0 | 36·2 | 38·5 | 41·1 | 44·1 | 46·2 |
| | Tot | 2·4 | 5·5 | 9·9 | 15·4 | 20·7 | 25·7 | 29·8 | 33·4 | 36·6 | 39·4 | 42·0 | 44·7 | 47·8 | 51·0 | 53·9 |
| Lymphomata etc. | M | 0·4 | 0·8 | 1·4 | 2·2 | 3·0 | 3·9 | 4·9 | 5·7 | 6·7 | 7·6 | 8·4 | 9·3 | 10·4 | 11·4 | 12·8 |
| | F | 0·3 | 0·5 | 1·0 | 1·2 | 1·6 | 2·0 | 2·5 | 2·9 | 3·2 | 3·6 | 3·7 | 4·1 | 4·6 | 5·4 | 5·8 |
| | Tot | 0·4 | 0·7 | 1·2 | 1·7 | 2·3 | 3·0 | 3·7 | 4·3 | 5·0 | 5·6 | 6·1 | 6·7 | 7·6 | 8·5 | 9·4 |
| Wilms' tumour | M | 0·5 | 1·2 | 2·3 | 3·3 | 4·1 | 4·7 | 5·1 | 5·3 | 5·4 | 5·6 | 5·7 | 5·7 | 5·7 | 5·8 | 5·8 |
| | F | 0·3 | 1·2 | 2·1 | 3·1 | 4·1 | 4·9 | 5·3 | 5·7 | 5·8 | 6·0 | 6·1 | 6·2 | 6·2 | 6·3 | 6·3 |
| | Tot | 0·4 | 1·2 | 2·2 | 3·2 | 4·1 | 4·8 | 5·2 | 5·5 | 5·6 | 5·8 | 5·9 | 5·9 | 6·0 | 6·0 | 6·0 |
| Malignant CNS tumours | M | 1·0 | 2·4 | 3·8 | 5·1 | 6·7 | 8·3 | 9·6 | 10·9 | 12·3 | 13·7 | 15·1 | 16·4 | 17·6 | 18·7 | 19·7 |
| | F | 0·9 | 2·0 | 3·2 | 4·3 | 5·5 | 6·8 | 7·9 | 8·9 | 10·0 | 11·2 | 12·1 | 12·8 | 13·6 | 14·5 | 15·2 |
| | Tot | 1·0 | 2·2 | 3·5 | 4·7 | 6·1 | 7·6 | 8·8 | 9·9 | 11·2 | 12·5 | 13·6 | 14·6 | 15·7 | 16·7 | 17·5 |
| Neuro- blastoma | M | 1·0 | 2·5 | 3·9 | 5·1 | 6·1 | 6·7 | 7·2 | 7·6 | 8·0 | 8·2 | 8·4 | 8·7 | 8·9 | 9·1 | 9·3 |
| | F | 1·2 | 2·1 | 3·3 | 4·1 | 4·7 | 5·4 | 5·7 | 6·0 | 6·2 | 6·3 | 6·6 | 6·9 | 7·1 | 7·3 | 7·4 |
| | Tot | 1·1 | 2·3 | 3·6 | 4·6 | 5·4 | 6·0 | 6·5 | 6·8 | 7·1 | 7·3 | 7·5 | 7·9 | 8·0 | 8·2 | 8·4 |
| Bone tumours | M | 0·0 | 0·0 | 0·1 | 0·1 | 0·2 | 0·3 | 0·4 | 0·6 | 0·9 | 1·3 | 1·5 | 1·9 | 2·2 | 2·8 | 3·8 |
| | F | 0·0 | 0·1 | 0·1 | 0·2 | 0·4 | 0·4 | 0·6 | 0·8 | 1·1 | 1·5 | 2·0 | 2·4 | 3·0 | 3·8 | 4·5 |
| | Tot | 0·0 | 0·0 | 0·1 | 0·2 | 0·3 | 0·4 | 0·5 | 0·7 | 1·0 | 1·4 | 1·7 | 2·2 | 2·6 | 3·3 | 4·1 |
| All solid tumours | M | 3·7 | 8·4 | 14·0 | 18·8 | 23·4 | 26·9 | 29·9 | 32·7 | 35·4 | 38·0 | 40·4 | 42·7 | 45·0 | 47·3 | 50·3 |
| | F | 3·8 | 8·1 | 12·7 | 16·8 | 20·3 | 23·7 | 26·2 | 28·5 | 30·6 | 32·9 | 35·2 | 37·2 | 39·2 | 41·9 | 44·5 |
| | Tot | 3·8 | 8·3 | 13·4 | 17·8 | 21·9 | 25·3 | 28·1 | 30·7 | 33·1 | 35·5 | 37·9 | 40·0 | 42·2 | 44·7 | 47·5 |
| All malignant tumours | M | 6·1 | 13·9 | 24·3 | 35·5 | 46·0 | 55·2 | 63·0 | 69·9 | 76·4 | 82·4 | 87·8 | 93·2 | 99·1 | 104·8 | 111·5 |
| | F | 6·1 | 13·7 | 22·2 | 30·9 | 39·0 | 46·7 | 52·6 | 57·9 | 62·5 | 66·9 | 71·4 | 75·7 | 80·3 | 86·0 | 90·6 |
| | Tot | 6·1 | 13·8 | 23·3 | 33·2 | 42·6 | 51·1 | 58·0 | 64·1 | 69·6 | 74·9 | 79·9 | 84·7 | 89·9 | 95·6 | 101·3 |

### RESULTS

### (1) *Variation of risk as between tumours*

Table II indicates the reporting of abdominal x-rays by case and control mothers according to the tumour developed by the case child. The numbers of such tumours are shown in the left hand column, followed by the expected and actual numbers of case children irradiated. (The " expected " numbers here are calculated on the basis of a uniform rate of x-raying amongst all tumour groups.) It will be seen that the agreement is very close.

In the next column are the numbers of control children irradiated in each group, followed, for completeness, by the numbers of pairs in which both case and control were exposed. Next come the relative risks estimated in the manner appropriate for matched pairs (Miettinen, 1970).

The highest relative risk estimates are for solid tumours and this disposes immediately of the notion that there is anything about radiation that is peculiar to leukaemia. The bone tumours form too small a group for us to be certain about their lower risk, though it is relevant here to observe that they appear later in life, either because of a longer latent period since initiation or because initiation was post natal. We must, in any case, bear in mind that we have singled out a particular group after looking at the data. In the absence of a prior hypothesis about specific tumours, the only valid procedure is to test the homogeneity of the nine groups as a whole; this homogeneity hypothesis is not rejected by a significance test.

Rather more precise estimates of the relative risk may be obtained by pooling the controls and using them all for each tumour group, though the estimates are on a slightly different basis since the pairs are no longer regarded as matched. Table II shows the alternative estimates obtained, together with lower and upper 95% confidence limits. This method assumes that the controls of children with different tumours are comparable with regard to their x-raying experience, which is in fact the case.

TABLE II.—*Numbers and Proportions of Mothers Irradiated by Tumour* (1953–67 *deaths; 8513 pairs*). *The Expected Number of Case Mothers X-rayed is Calculated on the Assumption of an Equal Frequency Among all Tumour Groups.* (*In this and Subsequent Tables " Both " Records Pairs in which Both Cases and Control were Irradiated; such Pairs are also Counted in the " Case " and " Control " Columns*)

| Tumour | No. of pairs | Expected no. x-rayed | No. of pairs in which mothers were irradiated: Case | Control | Both | R.R. | % cases x-rayed | Relative risk on basis of pooled controls: Estimate | 95% Limits |
|---|---|---|---|---|---|---|---|---|---|
| Leukaemias: | | | | | | | | | |
| Lymphatic | 2007 | 278 | 290 | 198 | **37** | 1·6 | 14·4 | 1·54 | 1·34 : 1·78 |
| Myeloid | 866 | 120 | 120 | 80 | **14** | 1·6 | 13·9 | 1·47 | 1·20 : 1·81 |
| Other/unspecified | 1179 | 164 | 159 | 128 | **16** | 1·3 | 13·5 | 1·43 | 1·19 : 1·71 |
| Lymphomata | 719 | 100 | 92 | 77 | **17** | 1·3 | 12·8 | 1·35 | 1·07 : 1·69 |
| All lymph/haemato-poietic | 4771 | 662 | 661 | 483 | **84** | 1·4 | 13·9 | 1·47 | 1·32 : 1·64 |
| Wilms' tumour | 590 | 82 | 87 | 47 | **6** | 2·0 | 14·7 | 1·59 | 1·25 : 2·01 |
| CNS tumours | 1332 | 185 | 179 | 138 | **19** | 1·3 | 13·4 | 1·42 | 1·20 : 1·69 |
| Neuroblastoma | 720 | 100 | 99 | 65 | **9** | 1·6 | 13·8 | 1·46 | 1·17 : 1·83 |
| Bone tumours | 244 | 34 | 26 | 22 | **4** | 1·2 | 10·7 | 1·11 | 0·74 : 1·66 |
| Other solid tumours | 856 | 119 | 129 | 85 | **18** | 1·7 | 15·1 | 1·63 | 1·33 : 1·98 |
| (All solid tumours) | 3742 | 519 | 520 | 357 | **56** | 1·5 | 13·9 | 1·47 | 1·31 : 1·66 |
| All malignant tumours | 8513 | 1181 | 1181 | 840 | **140** | 1·5 | 13·9 | 1·47 | 1·34 : 1·62 |

(2) *Variation in risk with age at death*

The Oxford Survey has been extended by degrees from the original age range of 0–9 years and questions arise as to whether the children affected later in life are suffering from radiogenic tumours and whether to include them in subsequent analyses. Table III shows the numbers of cases and controls x-rayed separately for haematopoietic and solid tumours and it will be seen that, although there appears to be some tailing off of the effect with age, the numbers are too small to be conclusive about this point. Certainly there is a case for extending the range beyond 9 years of age, but to select a cut-off point on the basis of this table is clearly to bias the resulting totals in favour of a case excess. It is on these grounds that we have decided to examine all ages up to and including 15 for further analyses.

Table III also shows the (cumulative) relative risks of being affected before the age specified, up to and including 9 years. The distributions have been cumulated in this way because interest in the relative risk of dying at a specified age is rather limited. These calculations are not permissible beyond the age of 10 years, since the one year ratios are based on smaller numbers of cases, *i.e.* those dying in the years 1961–67.

All the relative risk estimates in Table III are crude, in the sense that the cases contributing to each age estimate come from a different combination of birth years. Thus the crude estimates of age-specific risk are confounded with those specific to birth years. To get round this difficulty an analysis has been devised (see Bithell, 1975) which in effect simultaneously standardizes each factor for the other. The components of risk specific to particular years of birth and ages at death are estimated separately, and in practice it is wise to group the standardizing factor more broadly than the factor under study in order to cut down the number of parameters estimated. Thus, the single-year age-specific risks have been estimated while standardizing for year of birth in quinquennia. However, the resulting estimates have very wide confidence limits and show an erratic picture with no obvious trend and no consistency between solid and haematopoietic tumours. Indeed, with a likelihood ratio test it is not possible to reject the hypo-

TABLE III.—*Numbers of Cases and Controls Irradiated According to their Ages at Death and the Resulting Crude Relative Risks.* (1953–67 *deaths;* 8513 *pairs*)

| | Haematopoietic tumours | | | | | Solid tumours | | | | |
|---|---|---|---|---|---|---|---|---|---|---|
| | Mothers x-rayed | | | Rel. risk | | Mothers x-rayed | | | Rel. risk | |
| Age at death | Cases | Controls | Both | 1 year | Cum. | Cases | Controls | Both | 1 year | Cum. |
| 0 | 25 | 24 | **3** | 1·05 | 1·05 | 51 | 38 | **4** | 1·38 | 1·38 |
| 1 | 42 | 39 | **5** | 1·09 | 1·07 | 61 | 42 | **9** | 1·58 | 1·48 |
| 2 | 63 | 54 | **9** | 1·20 | 1·13 | 65 | 48 | **9** | 1·44 | 1·46 |
| 3 | 78 | 61 | **8** | 1·32 | 1·20 | 61 | 45 | **6** | 1·41 | 1·45 |
| 4 | 86 | 53 | **9** | 1·75 | 1·32 | 45 | 31 | **6** | 1·56 | 1·46 |
| 5 | 60 | 40 | **12** | 1·71 | 1·37 | 26 | 25 | **1** | 1·04 | 1·41 |
| 6 | 61 | 43 | **6** | 1·49 | 1·39 | 41 | 24 | **2** | 1·77 | 1·45 |
| 7 | 51 | 33 | **8** | 1·72 | 1·41 | 45 | 17 | **5** | 3·33 | 1·55 |
| 8 | 38 | 27 | **6** | 1·52 | 1·42 | 35 | 15 | **3** | 2·67 | 1·60 |
| 9 | 35 | 19 | **4** | 2·07 | 1·45 | 27 | 20 | **5** | 1·47 | 1·60 |
| 10 | 17 | 11 | **0** | 1·55 | — | 17 | 8 | **2** | 2·50 | — |
| 11 | 33 | 16 | **4** | 2·42 | — | 5 | 8 | **0** | 0·63 | — |
| 12 | 21 | 18 | **3** | 1·20 | — | 13 | 7 | **1** | 2·00 | — |
| 13 | 24 | 14 | **4** | 2·00 | — | 7 | 7 | **0** | 1·00 | — |
| 14 | 16 | 19 | **3** | 0·81 | — | 12 | 9 | **1** | 1·38 | — |
| 15 | 11 | 12 | **0** | 0·92 | — | 9 | 13 | **2** | 0·64 | — |
| All | 661 | 483 | **84** | 1·45 | — | 520 | 357 | **56** | 1·54 | — |

TABLE IV.—*Estimated Relative Risks of X-raying (with confidence limits, logarithms and their standard errors) for Different Ages at Death, Calculated according to the Methods outlined in Bithell (1975) and Standardized for Year of Birth in Quinquennia.* (8468 *pairs born since* 1943 *and with the index child dying under* 15)

| Age at death (years) | Haematopoietic tumours | | | | Solid tumours | | | | All tumours | | | |
|---|---|---|---|---|---|---|---|---|---|---|---|---|
| | | | Relative risk | | | | Relative risk | | | | Relative risk | |
| | Log rel. risk | S.E. | Est. | 95% limits | Log rel. risk | S.E. | Est. | 95% limits | Log rel. risk | S.E. | Est. | 95% limits |
| 0–2 | 0·288 | 0·15 | 1·33 | 0·99 : 1·81 | 0·589 | 0·15 | 1·80 | 1·35 : 2·41 | 0·464 | 0·11 | 1·59 | 1·29 : 1·96 |
| 3–5 | 0·575 | 0·12 | 1·78 | 1·39 : 2·27 | 0·340 | 0·15 | 1·40 | 1·05 : 1·88 | 0·484 | 0·09 | 1·62 | 1·35 : 1·95 |
| 6–8 | 0·496 | 0·15 | 1·64 | 1·24 : 2·19 | 0·765 | 0·18 | 2·15 | 1·51 : 3·06 | 0·604 | 0·11 | 1·83 | 1·47 : 2·28 |
| 9–11 | 0·635 | 0·20 | 1·89 | 1·26 : 2·82 | 0·242 | 0·25 | 1·27 | 0·78 : 2·08 | 0·468 | 0·16 | 1·60 | 1·17 : 2·17 |
| 12–14 | 0·033 | 0·23 | 1·03 | 0·66 : 1·61 | 0·245 | 0·30 | 1·28 | 0·71 : 2·30 | 0·081 | 0·18 | 1·08 | 0·76 : 1·54 |

thesis that the risk is the same for all ages. It seems wise, therefore—and certainly saving in space—to group the ages and Table IV shows the same analysis for the ages 0–14 grouped triennially. Again it will be seen that no very obvious picture emerges; it is, moreover, still not possible to detect differences between the age specific relative risks, and this remains true even with the broadest grouping into two groups, 0–9 years and 10–14 years.

It is therefore not easy with the present amount of information to be certain as to whether the relative risk does decline in the higher age groups, but certainly the figures obtained cast doubt on the picture emerging from MacMahon's study (based on much smaller numbers of cases) in which negligible risks were found after the age of 7.

It will be seen in (7) below that this analysis produces more interesting results when applied the other way round to the variations over birth year.

(3) *Epidemiological factors*

It is important to examine, and if necessary to standardize for, factors which could affect either the risk of being x-rayed or the inherent risk of malignancy, so that the possibility of spurious association may be excluded. This is the primary object of this section.

In the first instance, the effect of x-raying is quite consistent between the sexes, as is clearly shown by Table V; a $\chi^2$ test gives 0·23 with 1 d.f. MacMahon, too, found no significant difference in risk between the sexes.

TABLE V.—*Numbers of Case and Control Children Irradiated* in utero *by Sex. (Deaths* 1953–67; *malignant tumours:* 8513 *pairs)*

| | Males | Females | All |
|---|---|---|---|
| No. of pairs | 4823 | 3690 | 8513 |
| No. irradiated: | | | |
| Cases | 690 | 491 | 1181 |
| Controls | 483 | 357 | 840 |
| Both | 83 | 57 | 140 |
| Relative risk | 1·52 | 1·45 | 1·49 |
| Percent irradiated: | | | |
| Cases | 14·3 | 13·3 | 13·9 |
| Controls | 10·0 | 9·7 | 9·9 |

The birth rank order or sibship position, on the other hand, does appear to show an increasing risk for later born children (Table VI). In fact, although a $\chi^2$ test for the heterogeneity of the risk is not significant, fitting a linear trend-line does seem to indicate a positive relationship. Nevertheless, the importance of birth rank in Britain as a correlate of diagnostic x-raying is appreciably less important than in MacMahon's study, in which first and later pregnancies were x-rayed with population frequencies of 19·4% and 5·6% respectively.

It is also important to remember that sibship position is a variable that increases with maternal age, and Table VII shows that the latter variable gives a similar picture of increasing risk. When

TABLE VI.—*Numbers and Percentages of Case and Control Children Irradiated* in utero *According to the Sibship Position of the Index Child, and the Implied Relative Risks. The Relative Risks in the Last Row are Standardized Estimates*

| | Cases | | | Controls | | | Relative risk | |
|---|---|---|---|---|---|---|---|---|
| Position in family | X-rayed | All | % | X-rayed | All | % | Estimate | 95% limits |
| First | 547 | 3206 | 17·1 | 363 | 2767 | 13·1 | 1·36 | 1·18 : 1·57 |
| Second | 333 | 2637 | 12·6 | 245 | 2754 | 8·9 | 1·48 | 1·24 : 1·76 |
| Third | 147 | 1341 | 11·0 | 120 | 1476 | 8·1 | 1·39 | 1·08 : 1·79 |
| Fourth | 76 | 638 | 11·9 | 61 | 722 | 8·4 | 1·47 | 1·03 : 2·09 |
| Fifth | 33 | 303 | 10·9 | 24 | 357 | 6·7 | 1·71 | 0·99 : 2·95 |
| Later | 45 | 388 | 11·6 | 27 | 437 | 6·2 | 2·01 | 1·22 : 3·30 |
| All | 1181 | 8513 | 13·9 | 840 | 8513 | 9·9 | 1·44 | 1·31 : 1·58 |

TABLE VII.—*Numbers and Percentages of Cases and Controls Irradiated* in utero *Acording to Maternal Age* (*where known*) *and the Implied Relative Risks*

| Mother's age at birth of index child | Cases | | | Controls | | | Relative risk | |
|---|---|---|---|---|---|---|---|---|
| | X-rayed | All | % | X-rayed | All | % | Estimate | 95% limits |
| 15–19 | 43 | 301 | 14·3 | 21 | 181 | 11·6 | 1·28 | 0·74 : 2·24 |
| 20–24 | 284 | 2112 | 13·4 | 187 | 1880 | 9·9 | 1·41 | 1·16 : 1·71 |
| 25–29 | 360 | 2813 | 12·8 | 256 | 2818 | 9·1 | 1·47 | 1·24 : 1·74 |
| 30–34 | 260 | 1881 | 13·8 | 206 | 2137 | 9·6 | 1·50 | 1·24 : 1·83 |
| 35–39 | 163 | 1014 | 16·1 | 115 | 1134 | 10·1 | 1·70 | 1·32 : 2·19 |
| 40–44 | 60 | 314 | 19·1 | 50 | 336 | 14·9 | 1·35 | 0·90 : 2·04 |
| 45–54 | 7 | 33 | 21·2 | 4 | 19 | 21·1 | 1·06 | — |
| All known | 1177 | 8468 | 13·9 | 839 | 8505 | 9·9 | 1·48 | 1·34 : 1·62 |

both factors are taken together in 17 groups, however, a standardized estimate of 1·45 (95% confidence limits 1·32 : 1·60) was obtained, indicating that consideration of the second factor " explains " only a marginal extra amount of the observed excess risk (cf. the crude risk of 1·47).

Social factors also might affect the frequency of maternal irradiation. Table VIII shows the relative risk associated with x-raying in each social class group and also the idiopathic risk for a particular group relative to all others, calculated in respect of unirradiated mothers. (Social class is here defined in terms of the father's occupation, as indexed in the G.R.O. " Classification of Occupations ", 1960.) Although x-ray investigations certainly appear to be commoner for higher social class groups, no very clear picture emerges. There is no significant heterogeneity or linear trend in the x-raying risk, whose standardized estimate remains overwhelmingly significant.

The availability of treatment according to region of residence could also be of importance, but in this connection it should be remembered that cases and controls in the Oxford Survey were matched for region of residence at the time of death. Although migration will affect the issue somewhat, there is still a fair degree of matching for type of region during pregnancy. Moreover, as data on the place of birth are not readily available, it is not possible to carry out a more precise analysis. The situation is therefore that the type of region is unlikely to explain a significant component of the observed irradiation risk. Indeed, Table IX shows that the proportions of controls x-rayed and the relative risks are fairly uniform as between

TABLE VIII.—*X-raying of Case and Control Mothers According to Social Class. The Right-hand Columns show the Relative Risk due to X-raying in each Class and the Risk for Unirradiated Mothers in each Social Class Relative to All Others* (8513 *matched cases and controls*)

| Social class | Cases | | | Controls | | | Estimated relative risks associated with | |
|---|---|---|---|---|---|---|---|---|
| | X-rayed | All | % | X-rayed | All | % | X-raying | Social class |
| I | 61 | 384 | 15·9 | 30 | 282 | 10·6 | 1·60 | 1·36 |
| II | 191 | 1221 | 15·6 | 131 | 1168 | 11·2 | 1·47 | 1·05 |
| III | 708 | 5213 | 13·6 | 531 | 5313 | 10·0 | 1·42 | 0·96 |
| IV | 148 | 1084 | 13·7 | 92 | 1036 | 8·9 | 1·63 | 1·04 |
| V | 66 | 533 | 12·4 | 49 | 611 | 8·0 | 1·63 | 0·86 |
| Unknown/unemployed | 7 | 78 | 9·0 | 7 | 103 | 6·8 | 1·35 | — |
| All | 1181 | 8513 | 13·9 | 840 | 8513 | 9·9 | 1·47 | — |

TABLE IX.—*Numbers and Percentages of Cases and Controls Irradiated* in utero *According to the Type of Region of Residence and the Implied Relative Risks*

| Type of region | No. of pairs | No. of mothers irradiated: Cases | Controls | % | Both | Relative risk: Estimate | 95% limits |
|---|---|---|---|---|---|---|---|
| With conurbation | | | | | | | |
| County boroughs | 1160 | 157 | 129 | 11·1 | **20** | 1·25 | 0·98 : 1·61 |
| Municipal boroughs | 1229 | 203 | 121 | 9·8 | **17** | 1·81 | 1·43 : 2·31 |
| Urban districts | 358 | 57 | 28 | 7·8 | **4** | 2·25 | 1·40 : 3·63 |
| Without conurbation | | | | | | | |
| County boroughs | 1404 | 193 | 148 | 10·5 | **31** | 1·35 | 1·08 : 1·70 |
| Municipal boroughs | 1002 | 137 | 118 | 11·8 | **24** | 1·19 | 0·91 : 1·54 |
| Urban districts | 1600 | 226 | 147 | 9·2 | **30** | 1·63 | 1·31 : 2·03 |
| Rural | 1743 | 205 | 147 | 8·4 | **14** | 1·45 | 1·16 : 1·81 |
| No record/Other | 17 | 3 | 2 | 11·8 | **0** | 1·79 | — |
| All | 8513 | 1181 | 840 | 9·9 | **140** | 1·47 | 1·34 : 1·62 |

small and large towns, conurbations and country districts. The only significant associations in this table are a heterogeneity of x-raying frequency between types of region ($\chi^2 = 10{\cdot}3$, 3 d.f.) and a slight variation in risk with type of borough among conurbations, which does not seem to hold amongst non-conurbations.

A final possibility considered in this section is that x-rayed mothers may be atypical in that they have a higher susceptibility to illness in general, some of which may be associated with an incipient tumour in the foetus. The implication of this argument, which is similar to the one advanced by Fisher (1958) to "explain" the association between smoking and lung cancer in terms of a genetic predisposition, might be that the observed "risk" attributed to pre-natal irradiation is really the effect of selecting the high-risk pregnancies for radiography. Such arguments are notoriously difficult to refute but the Oxford Survey is in a position to examine the extent to which x-raying is associated with morbidity in this particular case. Table X shows the proportions of mothers x-rayed according to the number of illnesses recorded in the pregnancy. It can be seen that, as would be expected, there is indeed a strong association between this rather crude measure of morbidity and the degree of x-raying. Moreover, the risk of cancer among unirradiated mothers which is associated with having a specified number of illnesses

TABLE X.—*X-raying of Case and Control Mothers according to the Number of Illnesses Recorded for the Relevant Pregnancy. The Right-hand Columns show the Relative Risk due to X-raying in each Category and the Risk for Unirradiated Mothers of the Specified Number of Illnesses Relative to None* (8513 *matched cases and controls*)

| No. of illnesses | Cases: X-rayed | All | % | Controls: X-rayed | All | % | Estimated relative risks of malignant disease associated with: X-raying | Sickness |
|---|---|---|---|---|---|---|---|---|
| 0 | 639 | 5498 | 11·6 | 473 | 5885 | 8·0 | 1·51 | 1·00 |
| 1 | 374 | 2187 | 17·1 | 245 | 1979 | 12·4 | 1·46 | 1·16 |
| 2 | 118 | 627 | 18·8 | 94 | 515 | 18·3 | 1·04 | 1·35 |
| 3 | 50 | 201 | 24·9 | 28 | 134 | 20·9 | 1·26 | 1·58 |
| All | 1181 | 8513 | 13·9 | 840 | 8513 | 9·9 | 1·43* | — |

* Standardized estimate.

(relative to none being recorded) also increases strongly. The argument therefore stands up to some extent: different idiopathic and x-raying risks in different morbidity groups do partly explain the observed association. The bottom line of the Table, however, shows that the standardized risk estimate is still 1·43 and the variation of and trend in relative risk amongst the different groups is not significant ($\chi^2 = 5{\cdot}3$, 3 d.f.).

(4) *Dose-response relationship*

One of the most convincing pieces of supplementary evidence for the validity of the x-raying effect observed is the way in which it increases with the estimated exposure. Table XI shows the relative risks according to the hospital's record or estimate of how many films were exposed, where this is available, *i.e.* for around 60% of both cases and controls. Where several abdominal x-ray examinations were effected during the relevant pregnancy, details of the first investigation are analysed in this and subsequent sections. A straightforward test of the equality of the risks (excluding the unknown category) gives $\chi^2 = 11{\cdot}3$, 4 d.f., which is significant at the 5% level. But the trend is strongly upward with the number of films, and taking out the $\chi^2$ for trend gives much stronger evidence

TABLE XI.—*Numbers of Cases and Controls Irradiated* in utero *by the Probable Number of Exposures as Recorded by the Hospital*

| No. of films | Cases | Controls | Risk relative to non-irradiated cases: Estimate | 95% Limits |
|---|---|---|---|---|
| 1 | 287 | 239 | 1·26 | 1·06 : 1·50 |
| 2 | 199 | 154 | 1·35 | 1·09 : 1·67 |
| 3 | 96 | 65 | 1·54 | 1·13 : 2·11 |
| 4 | 59 | 28 | 2·18 | 1·40 : 3·41 |
| 5+ | 65 | 29 | 2·32 | 1·51 : 3·58 |
| Unknown | 475 | 325 | 1·53 | 1·32 : 1·77 |
| All cases | 1181 | 840 | 1·47 | 1·34 : 1·62 |

FIG. 1.—Variation in risk of obstetric x-raying according to the hospital record of the number of films exposed. The " excess risk " is defined as relative risk −1; the vertical brackets indicate the 95% confidence intervals.

of a dose-response relationship, namely $\chi^2 = 10{\cdot}5$, 1 d.f.

In view of the great difficulty of obtaining even an approximate idea of the number of films and of the variations in dose per exposure, both in time and across different hospitals, it is remarkable that the effect stands out as clearly as it does.

There are a number of ways of analysing the exposure data to obtain a quantitative relationship. Figure 1, for example, shows the result of plotting the excess risk against the number of films and fitting a linear weighted regression. (The risks in this analysis were computed as independent estimates, exploiting the matching by eliminating pairs where both case and control were x-rayed.) It will be seen that the regression line passes virtually through the origin (the intercept being $0{\cdot}066 \pm 0{\cdot}12$) and has a slope of $0{\cdot}180 \pm 0{\cdot}06$. If we constrain the curve to go through the origin and take logs we can estimate the degree, or power-law index, of the relationship as $1{\cdot}06 \pm 0{\cdot}27$, a result not very different from the $0{\cdot}915 \pm 0{\cdot}329$ obtained by Stewart and Kneale (1970) in a more extensive analysis that allowed for the variation of exposure with time. The evidence is therefore very convincing that the relationship between risk and the number of films is linear.

Other authors have adopted different approaches. Newcombe and McGregor (1971), for instance, argue in effect that because of the width of the confidence intervals for the risk estimates, relationships other than the linear cannot be precluded. Holford (1974), however, counters this point of view by an analysis based on the statistical concept of "support" for a hypothesis and concludes what might be expected from Fig. 1, that the linear hypothesis is much the most "likely". The more classic theory of significance testing gives essentially the same result, *i.e.* fitting a quadratic curve gives a fit which is not significantly better.

Attempts to estimate the radiation dose per film and so devise a genuine dose-response curve are frustrated by the shortage of reliable information and the fact that improvements in obstetric techniques have probably led to marked reductions in dose per film over the period of the study. The calculations of Stewart and Kneale (1970) are based on the assumption that the mean foetal dose per film declined from 460 mrad in 1945 to around 200 mrad in 1965. This led to an estimated risk of $572 \pm 133$ cases per million foetal rad. Considerable controversy has been aroused by the apparent incompatibility of this estimate with those obtained from studies of the survivors of the Hiroshima and Nagasaki bombing (Jablon and Kato, 1970). Follow-up to the age of 10 years revealed only one case of malignant disease in children *in utero* at the time of the explosions, and this is several times smaller than would be predicted by extrapolating the Oxford Survey estimate on a linear dose-response line.

However, it is clear that the statistical error in the latter estimate is unimportant beside the uncertainty about the actual dosage in ante-natal diagnostic radiography. Mole (1974) reviews the situation and concludes that "the upper limit of the risk derived from Japanese data for bomb irradiation can then be regarded as compatible with the estimates for diagnostic radiography but only if all the assumptions in the calculations are chosen with that intent". Moreover, he then questions these assumptions in the light of radiobiological knowledge about cell sterilization and the resultant non-linearity of the dose-response curve.

At all events, the biological insult resulting from the Japanese explosions was so different in character from the effect of diagnostic radiography that it requires a formidable set of assumptions to infer from the absence of a small number of Japanese cases that the very reasonable results of the Oxford Survey are entirely artefactual.

TABLE XII.—*Numbers of Cases and Controls Irradiated* in utero *according to the Month or Trimester of Pregnancy of the Exposure, as Recorded by the Hospital*

| | Cases | Controls | Risk relative to non-irradiated cases: Estimate | Risk relative to non-irradiated cases: 95% limits | Mean no. of films per case |
|---|---|---|---|---|---|
| Month: | | | | | |
| First | 11 | 0 | — | 2·51 : — | 4·6 |
| Second | 11 | 0 | — | 2·51 : — | 5·2 |
| Third | 16 | 4 | 3·84 | 1·41 : 10·4 | 4·6 |
| Trimester: | | | | | |
| First | 38 | 4 | 8·95 | 3·53 : 22·7 | 4·78 |
| Second | 61 | 51 | 1·25 | 0·86 : 1·81 | 3·24 |
| Third | 700 | 521 | 1·41 | 1·25 : 1·58 | 2·06 |
| Unknown | 382 | 264 | 1·51 | 1·29 : 1·78 | — |
| All cases | 1181 | 840 | 1·47 | 1·34 : 1·62 | 2·27 |

(5) *Timing of the x-ray investigations*

Table XII shows the stages of pregnancy in which the exposures were made, according to the hospital records. Again the proportions of known dates are very similar for cases and controls—a little under 70% in each. The early months appear to carry a much higher risk, though the actual numbers x-rayed then are very small. (It is not possible to estimate the risk for the first 2 months as no controls enter the appropriate cells. A lower limit of risk can, however, be estimated as 2·51.) A $\chi^2$ test for the comparability of the 3 trimesters is of course highly significant (19·0 with 2 d.f.); this is due entirely to the first trimester, the difference between the second and third not being statistically significant. It will be seen from the last column of Table XII, however, that the differences in risk estimates are at least partly due to larger exposures.

It is noteworthy that 2 of the 38 cases x-rayed in the first trimester developed tumours of the female genital organs. These occur generally as only 0·6% of all tumours in children so that the expected number out of 38 would have been only 0·23. This is a highly selected item of information, however, and a general test over the whole range of tumours and stages of pregnancy shows no association between them.

(6) *Reason for the investigation*

The reasons for the diagnostic x-rays were ascertained both from the mothers' interviews and also from the hospitals' records, which were available for about 65% of both cases and controls. The great majority of the examinations were for obstetric reasons, as may be seen from Table XIII. It should be remembered that actual twins are excluded from this table so that the first row (suspected twins) applies to singleton births. At first sight the relative risk of 1·14 in this row appears to be very low, but reference to the last column shows that this reason for x-ray involves only 1·5 films per case on average and the observed risk is not significantly less than would be predicted by the figures in Table XI for this dosage. Table XIII also explains in terms of dose the higher risks associated with pelvimetry and certain non-obstetric investigations; in addition, one can see that the latter take place more frequently in the first trimester, with its high associated risk (Table XII). The hyperemesis category appears to have a particularly high risk. Typically, such patients were investigated by barium meals or cholecystograms; the probability is that these were all carried out before pregnancy was suspected.

A $\chi^2$ test of the risks in Table XIII

TABLE XIII.—*Relative Risk according to Indication for X-ray (as given in Hospital Records) together with Numbers in First Trimester and Mean Number of Films*

| Indication for x-ray | Cases No. | Cases % of known | Controls No. | Controls % of known | Relative risk Estimate | Relative risk 95% limits | No. x-rayed in first trimester | Mean no. of films per case x-rayed |
|---|---|---|---|---|---|---|---|---|
| Obstetric: | | | | | | | | |
| Suspected twins* | 179 | 23·6 | 165 | 30·2 | 1·14 | 0·92 : 1·41 | 2 | 1·51 |
| Suspected abnormal position | 225 | 29·6 | 167 | 30·6 | 1·41 | 1·15 : 1·72 | 0 | 1·88 |
| Routine pelvimetry | 90 | 11·9 | 38 | 7·0 | 2·46 | 1·69 : 3·58 | 3 | 3·11 |
| Other obstetric | 202 | 26·6 | 151 | 27·7 | 1·40 | 1·13 : 1·73 | 2 | 2·40 |
| All obstetric | 696 | 91·7 | 521 | 95·4 | 1·40 | 1·24 : 1·57 | 7 | 2·11 |
| Other abdominal: | | | | | | | | |
| Intravenous pyelogram | 10 | 1·3 | 5 | 0·9 | 2·00 | 0·74 : 5·41 | 3 | 6·30 |
| Hyperemesis | 9 | 1·2 | 3 | 0·5 | 2·84 | 0·89 : 9·06 | 8 | 4·75 |
| Injury to mother | 44 | 5·8 | 17 | 3·1 | 2·66 | 1·54 : 4·60 | 19 | 4·03 |
| All known indications | 759 | 100 | 546 | 100 | 1·45 | 1·30 : 1·63 | 37 | 2·32 |
| Indications not known | 422 | 36% (of all) | 294 | 35% (of all) | 1·50 | 1·29 : 1·75 | 1 | — |
| All x-rayed | 1181 | | 840 | | 1·47 | 1·34 : 1·62 | 38 | 2·27 |

* Pairs where case or control actually was a twin are excluded.

gives 20·0 with 6 d.f. and indicates highly significant variation.

(7) *Date of birth*

As discussed in (2) above, it is necessary to take account of the different ages represented when analysing the cohort or date of birth effect. The same analysis as was used for the age effect was used to standardize for age in five 3-year groups with the results shown in Fig. 2. The graphs show a remarkable consistency between the haematopoietic and solid tumours and the overall picture is clearly one of declining risk. An interesting possibility is that there was a remission in this general decline in the mid-fifties but although the effect looks genuine, the eye is deceived by the correlations between the time points. (This effectively means that there is less information and more variability than if the observations were independent and consequently an effect which looks statistically significant may in fact not be so.) A weighted regression does in fact suggest that the rise is not significant and the linear trend-line shown in Fig. 2(b) gives an adequate fit to the data. That is not to say that it is satisfactory for extrapolation, since it predicts a negative risk due to x-raying after 1967. But it appears at the moment that the risk has diminished to the point where it might be quite difficult to detect in the future.

The general picture of varying risks shown by Fig. 2 accords well with the results of Stewart and Kneale (1968, Fig. 2) obtained by an entirely different method. The present analysis extends the range of birth years considered and, of course, includes the older children omitted from the earlier calculations.

It is interesting to examine possible reasons for the decline in risk, and two obvious possibilities arise. In the first place, the radiation dose per film exposed has probably declined over the years in Great Britain (Stewart and Kneale, 1970), though some doubt persists about whether American experience is as favourable (Landau and Stewart, 1974). Secondly, the actual number of film exposures per investigation has declined significantly (see Fig. 3(a)).

Thus the controls, for example, show a decline of 0·075 exposures per case

FIG. 2.—Estimates of log relative risk by birth cohort, standardized for age in quinquennia: (a) solid and haematopoietic tumours separately; (b) all tumours, with a weighted regression fitted.

per annum in a regression which gives a highly significant F-ratio of 28 with 1 and 9 d.f.

Finally, Fig. 3(b) shows the frequency of obstetric x-raying over the years. These estimates are confounded with any possible age effect—due, for example, to a memory factor—in exactly the same way as the estimates of relative risk. However, Kneale (1971) has shown that age at death has remarkably little effect on the frequency of x-ray reporting, while the differences between the birth cohorts are highly significant. His analysis applies only to deaths under 10 years of age before 1966; it is still too early to assess the rates since then, and the sharp increase to 16% shown in Fig. 3(b) may well be a result of sampling fluctuation.

### DISCUSSION

As has been intimated above, it is logically impossible to conclude for certain from epidemiological observations that a particular association is causal in a specified direction. It is also true that a retrospective study inevitably involves difficulties not shared by prospective investigations. That is not to say that the retrospective study is worthless for it has enormous potential in terms of information.

What we have tried to do in this paper is to present the Oxford data—unique as they are—fairly and systematically. It is true that when the risks are standardized for various epidemiological factors a small reduction in the estimates results; yet this is quite insufficient to " explain " the radiation association completely. Moreover, as pointed out by Mole (1974), the fact that the excess mortality in twins is very similar to that in singletons, despite the very different irradiation rates, militates strongly against

(a)

(b)

Fig. 3.—Trends in x-raying as estimated from data on the control children: (a) mean number of films per investigation; (b) proportion of foetuses irradiated.

the possibility of the x-ray excess being due to selection of pre-disposed cases for x-raying.

A genuine causation would be more convincing and perhaps more in line with prior expectations if the irradiated cases exhibited more differences from the unirradiated, especially as regards age and tumour type. Yet the absence of such differences is not inherently detrimental to the radiation risk hypothesis, especially if we surmise that the great majority of childhood tumours, as yet unexplained in aetiological terms, are initiated *in utero* by a process similar to the molecular effects of low-level radiation.

Moreover, against any doubts about the carcinogenic effect of obstetric radiography may be set the extremely plausible relationship between the risk and the number of films. Indeed, all the attributes of the irradiation process—the reason, the timing and the year, as well as the presumed dose—give much convincing supplementary support to the radiation-risk hypothesis, being as they are so well in accord with prediction. Certainly a complete explanation of the Oxford data in other terms would need a most elaborate and ingenious set of assumptions.

Finally, it may be remarked that the simplest model of radio-carcinogenesis —that of a Poisson process representing discrete cellular accidents—would definitely predict a low-level effect of irradiation. The existence of a threshold requires altogether more extensive assumptions, incorporating the ideas not only of multiple-hit processes but also of repair capability. One is therefore in the usual Occam's razor situation of accepting not so much a final proof as the simplest and most obvious implication: that any radiation is potentially carcinogenic and that the risk per rad for *in utero* exposure is roughly in line with that implied by the Oxford Survey.

The Oxford Survey of Childhood Cancers was supported during the preparation of this paper by the U.S. Public Health Service (Grant No. 12208 and Contract No. FDA 72–126), the Medical Research Council (Grant No. G.964/230/C), and the Marie Curie Memorial Foundation.

The data were collected by doctors on the staff of all County and County Borough Health Departments in England, Scotland and Wales. The authors wish to express their gratitude to the many people who have contributed to the conduct and analysis of the Survey, but particularly their colleague, G. J. Draper, who has been a continual source of guidance on the interpretation of the results.

### REFERENCES

BITHELL, J. F. (1975) Using the Logistic Model to Standardise Relative Risk Estimates in Unbalanced Tables. *Biometrics*. Submitted for publication.

COURT BROWN, W. M., DOLL, R. & HILL, A. B. (1960) Incidence of Leukaemia after Exposure to Diagnostic Radiation *in utero*. *Br. med. J.*, ii, 1539.

DIAMOND, E. L., SCHMERLER, H. & LILIENFELD, A. M. (1973) The Relationship of Intra-uterine Radiation to Subsequent Mortality and Development of Leukèmia in Children. *Am. J. Epidem.*, **97**, 283.

FISHER, R. A. (1958) Cancer and Smoking. *Nature, Lond.*, **182**, 596.

HEWITT, D., SANDERS, B. M. & STEWART, A. M. (1966) Oxford Survey of Childhood Cancers: Progress Report IV—Reliability of Data Reported by Case and Control Mothers. *Mth. Bull. Minist. Hlth Lab. Serv.*, **25**, 80.

HOLFORD, R. M. (1974) The Relation between Juvenile Cancer and Obstetric Radiography. Personal communication.

JABLON, S. & KATO, H. (1970) Childhood Cancer in Relation to Prenatal Exposure to Atomic-Bomb Radiation. *Lancet*, ii, 1000.

KNEALE, G. W. (1971) Problems arising in Estimating from Retrospective Survey Data the Latent Periods of Juvenile Cancers Initiated by Obstetric Radiography. *Biometrics*, **27**, 563.

LANDAU, E. & STEWART, A. (1974) X-rays, Influenza and Leukemia: and an Examination of the Oxford Study of Childhood Cancers. *Proc. Fifth Int. Congr. Radiat. Res.*, Seattle, Washington.

MACMAHON, B. (1962) Prenatal X-ray Exposure and Childhood Cancer. *J. natn. Cancer Inst.*, **28**, 1173.

MIETTINEN, O. S. (1970) Estimation of Relative Risk from Individually Matched Series. *Biometrics*, **26**, 75.

MOLE, R. H. (1974) Antenatal Irradiation and Childhood Cancer: Causation or Coincidence? *Br. J. Cancer*, **30**, 199.

NEWCOMBE, H. B. & MCGREGOR, J. F. (1971) Childhood Cancer Following Obstetric Radiography. *Lancet*, ii, 1151.
REGISTRAR GENERAL (1960) *Classification of Occupations*. London: H.M.S.O.
STEWART, A. M., WEBB, J. W., GILES, B. D. & HEWITT, D. (1956) Preliminary Communication: Malignant Disease in Childhood and Diagnostic Irradiation *in utero*. *Lancet*, ii, 447.
STEWART, A. M., WEBB, J. W. & HEWITT, D. (1958) A Survey of Childhood Malignancies. *Br. med. J.*, i, 1495.
STEWART, A. M. & KNEALE, G. W. (1968) Changes in the Cancer Risk associated with Obstetric Radiography. *Lancet*, i, 104.
STEWART, A. M. & KNEALE, G. W. (1970) Radiation Dose Effects in Relation to Obstetric X-rays and Childhood Cancers. *Lancet*, i, 1185.
STEWART, A. M. (1973) Cancer as a Cause of Abortions and Stillbirths: the Effect of these Early Deaths on the Recognition of Radiogenic Leukaemias. *Br. J. Cancer*, **27**, 465.